# Retrospect of the Medical Sciences

**Published:** 1843-09-02

**Authors:** 


DR. RICORD ON THE TREATMENT OF GONORRHŒA.

We may class the treatment under five heads: 1, the prophylactic; 2, the abortive; 3, the treatment of the acute stage; 4, of the declining period; and 5, of the chronic form.

1. *Prophylactic Treatment.*—The first precept is, doubtless, never to expose oneself to contagion. Nicholas] Massa, states, with admirable *naïveté*, that it is absolute madness to court danger of this description. I have remarked that gonorrhœa is seldom the result of one single connexion—on the contrary, it will be observed in almost all cases that it has followed the repetition within a short time of venereal pleasures. N. Massa also lays down as a rule, to avoid prolonged coitus, which determines and prediposes to the invasion of the disease.

Lotions, with cold water, will be found also an advantageous preparation, but tepid washes and warm baths I consider as the very worst means of prevention of the disease, as they produce a degree of congestion highly predisposing to subsequent inflammation.

I also reject absolutely, medicinal lotions, which in general irritate in a useless manner organs undergoing a physiological excitement. On the contrary, emission of urine immediately after connexion should on no occasion be neglected. It is a natural injection, from the bladder forwards, which washes off any improper stimulus which may have found its way into the urethra.

Other prophylactic measures have been advocated, mechanically preventing actual contact, and thus the contraction of the disease.

2. *Abortive Treatment.*—It is indispensable to proceed to the abortive treatment when the disease is not too far advanced. It is an erroneous, but generally received notion, that it is useful to allow the secretion to continue a certain time before any attempts are made to arrest its progress. No possible advantage can result from the prolongation of an inflammation on any mucous membrane, much less the urethra. The consequence of the unfortunate doctrine we allude to is, to render the discharge more difficult of cure by permitting the mucous membrane to become altered in its structure by the chronic establishment of an inflammation, so as to promote strictures, abscess, softening, &c. Such a doctrine tends to make epididymitis and other complications of gonorrhœa more frequent, and to increase the sum of human suffering, whilst the chances of bad results without any compensating benefit exist in the fullest degree. We strongly recommend the adoption of the abortive treatment.

3. *Treatment of the Acute Stage.*—Here the antiphlogistic regimen, local and sometimes general bleeding, revulsives on the intestinal tube, diluent drinks, baths, and antispasmodics find their full application. We shall, at a subsequent period, refer to this treatment in detail.

4. *Treatment of Declining Stage.*—The indications are precisely the same as in the abortive treatment, for whilst in the incipient stage inflammation has not yet set in, here it has already ceased to manifest itself by its acute symptoms.

5. *Treatment of Chronic Stage.*—In order properly to treat this period, it is to be recollected that gleet generally is indebted for its existence to some local or general cause which keeps up the irritation, and which must be discovered, in order that success may attend the treatment. The running is mostly symptomatic of another disease, towards which the attention of the surgeon should be directed.

It has been proposed by some, and it still is the object of the practice of many, to obtain a renewal of the original discharge by exciting the urethra with the passage of simple catheters, or of instruments covered with various stimulant substances. This practice I condemn, as the surgeon acts blindfolded. The only general precept to be laid down is to endeavour to acquire by proper investigation accurate knowledge of the state to which chronic inflammation has brought the membrane, and to guide the subsequent treatment thereon.

We now begin the study of each particular variety of gonorrhœa. It will be much shortened by the foregoing general observation, but important precepts of diagnosis and treatment arise out of the consideration of the various kinds of discharge. We deem it advisable to examine gonorrhœa:—1, as it occurs exclusively in the male, as in external gonorrhœa; 2, in the female, in the urethra, the external organs of generation, the vagina, the uterus, either separately affected or in combination; 3, in both sexes, such as blenorrhagia of the eye, the anus, various folds of the skin, the nose, the ear, or mouth.—*Prov. Med. Journ.*

---

DR. RICORD ON EXTERNAL GONORRHŒA.

External gonorrhœa—chaudepisse bâtarde—balanitis—posthite—balanoposthite.

The causes which produce more particularly this form are phymosis, congenital or acquired, the accumulation of natural secretions, the habit of masturbation.

*Premonitory Signs.*—A sensation of weight or uneasiness, an unusual degree of heat about the part, a distressing sense of itching, are the only signs referable to the early stage of the disease.

*Symptoms.*—Inflammation may extend to the whole lining of the prepuce, or be limited to a small portion of mucous surface. The membrane soon becomes red from injection, its natural secretion is exchanged for a discharge thicker and more creamy than that of uretritis, and incarceration within the prepuce, together with incipient decomposition, communicate to this liquid a particularly offensive and irritating character. Contact is painful, desquamation of the epithelium, rendering the organ exquisitely sensitive from the denudation of a number of nervous papillæ.

*Complications.*—It is not uncommon to find œdema of the lax subcutaneous cellular structure complicating the disease. Erysipelas is occasionally met with, doubling the size of the penis, and not unfrequently acquiring so much violence as to threaten or even produce gangrene.

But in contradistinction to urethral blenorrhagis, epididymitis is never the consequence of external gonorrhœa, and very seldom bubo.

Eczema, on the other hand, and herpes break out on the glans, stimulated into existence by the presence of the inflammation.

Condylomata and excrescences of various kinds are the result, and may become the cause of balanoposthitis.

Chancre and mucous tubercle may also complicate or |produce external gonorrhœa. It is not a little remarkable that the first of these affections gives rise in its vicinity to a perfectly simple inflammation, the secretions of which may be inoculated with impnnity, whereas pus from the neighbouring chancre will *constantly and at the same time* give a specific sore by inoculation.

*Differential Diagnosis.*—From eczema or herpes præputialis, external gonorrhœa will be easily distinguished by the diffused character of the inflammation, the absence of vesicles of any kind during its first period, or of those small circular erosions which follow herpes, and which have more analogy of appearance with superficial chancre than with simple inflammation of the prepuce or glans.

The scalding in passing water concentrated in the fossa navicularis, a view of the discharge coming from the urethra in cases where the foreskin can be slightly drawn back, also the particular pain caused by the distension of the urethra during erection, will suffice to establish the diagnosis between blenorrhagia and balano-posthitis, in which erections are free from uneasiness; pressure determines pain *around the base* of the glans, and the discharge comes from the cul de sac formed by the reflection of the mucous membrane.

As to chancre, it will be sometimes possible to obtain a rational diagnosis of its existence under an irreducible phymosis, from a certain degree of painful hardness in one particular spot of the corona glandis, from the presence of enlarged ganglions in the groins, rather uncommon in simple balinitis; but one sign only can be implicitly relied upon to distinguish absolutely simple external gonorrhœa from phymosis complicated with chancre, and that sign is inoculation with the lancet.—*Ibid.*

---

*Pathological Researches into the Local Causes of Deafness, based on one Hundred and Twenty Dissections of the Human Ear.* By JOSEPH TOYNBEE, F. R. S., Surgeon to the St. George's and St. James' Dispensary.

The researches of which this is a summary view, are in continuation of a previous paper contained in the twenty-fourth volume of the Society's Transactions. The principal practical conclusion to which they lead is, that the most prevalent cause of deafness is chronic inflammation of the mucous membrane which lines the tympanitic cavity, and that by far the greater majority of cases commonly called nervous deafness ought more properly to be attributed to this cause.

The pathological conditions to which inflammation of the mucous membrane gives rise are divided in the paper into three stages.

*In the first stage* the membrane retains its natural delicacy of structure, though its blood-vessels are considerably enlarged and contorted; blood is effused into its substance, or more frequently at its attached surface; blood has also been found between the membrane and the membrane of the fenestra rotunda, and in very acute cases lymph is effused over its free surface.

The second stage is characterised by the following pathological conditions :—

1st. The membrane is very thick, and often flocculent. In this state the tympanitic plexus of nerves becomes concealed, the base and crura of the stapes are frequently entirely imbedded in it, while the fenestra rotunda appears only like a superficial depression in the swollen membrane.

2nd. Concretions of various kinds are visible on the surface of the thickened membrane. In some cases these have the consistence of cheese, and are analogous to tuberculous matter; in others they are fibro-calcareous, and exceedingly hard.

3rd. But by far the most frequent and peculiar characteristic of this second stage of the disease is, the formation of membranous bands between various parts of the tympanitic cavity. These bands are at times so numerous as to occupy nearly the entire cavity; sometimes they connect the inner surface of the membrana tympani to the internal wall of the tympanum, to the stapes and to the incus. They have also been detected between the malleus and the promontory, as well as between the incus, the walls of the tympanum, and the sheath of the tensor tympani muscle, as well as between various parts of the circumference of the fenestra rotunda. But the place where the adhesions are most frequently visible is between the crura of the stapes and the adjoining walls of the tympanitic cavity; this was the case in twenty-four instances out of a hundred and twenty dissections, being a fifth of the number. These bands of adhesion sometimes contain blood and scrofulous matter.

*In the third stage* of inflammation of the membrane it becomes ulcerated; the membrana tympani is destroyed, and the tensor tympani muscle is atrophied. The ossicula auditus are diseased, and ultimately discharged from the ear, and the disease not unfrequently communicates itself to the tympanic walls, affecting also the brain and other important organs.

The following is a tabular view of the mucous membrane of the the tympanic cavity in the 120 dissections related in this paper.

*In the first stage of inflammation.*

| | | |
|---|---|---|
| 1. | With simple inflammation of the membrane, its vessels being enlarged, tortuous, and distended with blood - - - - | 10 |
| 2. | Ditto, with an accumulation of mucous - | 1 |
| 3. | Membrane inflamed, with effusion of blood into its substance - - - - - | 3 |
| 4. | Membrane inflamed, with effusion of serum, tinged with blood, into the tympanic cavity - - - - - - - | 1 |
| 5. | Membrane inflamed, with lymph effused into the tympanic cavity - - - - | 2 |
| 6. | Membrane inflamed, with blood and lymph effused into the tympanic cavity - - | 2 |
| 7. | Membrane inflamed, with effusion of pus into the tympanic cavity - - - - | 1 |

*Dissections illustrative of the second stage of inflammation.*

| | | |
|---|---|---|
| 1. | With simple thickening of the lining membrane - - - - - - - - | 5 |
| 2. | The membrane thick and pulpy - - | 2 |
| 3. | Ditto, ditto, and the cavity full of bands of adhesion - - - - - - - - | 1 |
| 4. | The membrane thick and flocculent - • | 1 |
| 5. | Membranous bands connecting the membrana tympani to the inner wall of the tympanum - - - - - - | 5 |
| 6. | Membranous band connecting the membrana tympani to the promontory and the chorda tympani to stapes - . - | 1 |
| 7. | Membranous bands connecting the membrana tympani to the incus - - - - | 1 |
| 8. | Ditto ditto to the stapes - - - | 2 |
| 9. | Ditto connecting the membrana tympani and chorda tympani nerve to the stapes - | 1 |
| 10. | Ditto connecting the membrana tympani and malleus to the promontory - - | 1 |
| 11. | Ditto connecting the membrana tympani to the incus - - - - - - - - | 2 |
| 12. | Ditto connecting the membrana tympani and assecles to the inner wall of the tympanum - - - - - - | 1 |

13. Ditto connecting the malleus to the inner wall of the tympanum - - - 2
14. Ditto connecting the incus to the inner wall of the tympanum - - - - - 1
15. Ditto connecting the stapes to the promontory - - - - - - - 24
16. Anchylosis of the stapes to the fenestra ovalis - - - - - - - - 2
17. Membranous bands, forming a network over the fenestra rotunda - - - - - 2
18. A broad membrane passing from the promontory to the mastoid cells - - 2
19. The cavity of the tympanum full of bands of adhesions - - - - - - 1
20. Membranous bands containing scrofulous matter - - - - - - - - 3
21. The cavity of the tympanum full of calcareous concretion - - - - - - 4
22. Ditto, full of caseous concretion - - 2
23. With ridges of bone projecting from the surface of the promontory - - - 2

*Dissections illustrative of the third stage of inflammation.*

1. With ulceration and thickening of the mucous membrane, attended by the formation of pus - - - - - - 3
2. With the ulceration of the membrane, and loss of one or more of the ossicula - 3

It thus appears that of the 120 dissections there were—

20 Specimens in the first stage of inflammation of the tympanic cavity.
65 Ditto in the second stage.
6 Ditto in the third.
29 Ditto in a healthy state.

120

*Lond. Med. Gaz.*

PLEA OF INSANITY IN CRIMINAL CASES.

The report of the judges has recently been made to the House of Lords, in answer to the several questions proposed to them relative to the legal application of the plea of insanity in criminal cases; and as this is, perhaps, one of the most difficult as well as unsatisfactory points upon which professional evidence is required in courts of law, it was hoped that the result of the inquiry would be the construction of more direct and certain rules for the guidance of medical witnesses than at present exist. How far such expectation has been fulfilled we shall hereafter endeavour to determine. The questions and the answers returned to them are the following:—

"*Question* 1.—What is the law respecting alleged crimes committed by persons afflicted with insane delusion, in respect of one or more particular subjects—as, for instance, when at the time of the commission of the alleged crime, the accused knew he was acting contrary to law, but did the act complained of with the view, under the influence of some insane delusion, of redressing or avenging some supposed grievance or injury, or of producing some supposed public benefit.

"*Answer.*—The opinion of the judges was that, notwithstanding the party committed a wrong act while labouring under the idea that he was redressing a supposed grievance or injury, or under the impression of obtaining some public or private benefit, he was liable to punishment.

"*Q.* 2,—What are the proper questions to be submitted to the jury when a person, alleged to be afflicted with insane delusions respecting one or more particular subjects or persons, is charged with the commission of a crime—murder, for example—and insanity is set up as a defence?

"*A.*—The jury ought in all cases to be told that every man should be considered of sane mind until the contrary were clearly proved in evidence. That before a plea of insanity should be allowed, undoubted evidence ought to be adduced that the accused was of diseased mind, and that at the time he committed the act he was not conscious of right or wrong. This opinion related to every case in which a party was charged with an illegal act, and a plea of insanity was set up. Every person was supposed to know what the law was, and, therefore, nothing could justify a wrong act unless it was clearly proved that the party did not know right from wrong. If that was not satisfactorily proved, the accused was liable to punishment; and it was the duty of the Judges so to tell the jury when summing up the evidence, accompanied by those remarks and observations which the nature and peculiarities of each case might suggest and require.

"*Q.* 3.—In what terms ought the question to be left to the jury as to the prisoner's state of mind at the time the act was committed?

"This question was not answered.

"*Q.* 4.—If a person, under an insane delusion as to existing facts, commit an offence in consequence thereof, is he thereby excused?

"*A.*—If the delusion were only partial, the party accused was equally liable with a person of sane mind; if the accused killed another in self-defence, he would be entitled to an acquittal; but if the crime were committed for any supposed injury, he would then be liable to the punishment awarded by the laws to his crime.

"*Q.* 5.—Can a medical man, conversant with the disease of insanity, who never saw the prisoner previously to the trial, but who was present during the whole trial and the examination of all the witnesses, be asked his opinion as to the state of the prisoner's mind at the time of the commission of the alleged crime, or his opinion whether the prisoner was conscious at the time of doing the act that he was acting contrary to law, or whether he was labouring under any, and what delusion at the time?

"*A.*—The question could not be put in the precise form stated above, for by doing so it would be assumed that the facts had been proved. When the facts were proved and admitted, then the question—as one of science—would be generally put to a witness under the circumstances stated in the interrogatory."

Such at present is the opinion of the chief members of the legal profession on the subject of insanity, when urged as the defence of criminal acts—such are the laws by which the question of personal responsibility is to be determined. It would be far from an unprofitable inquiry to ascertain the results which the application of these views to former judicial investigations would produce. Many previous decisions, and some of those of modern date, would be found altogether at variance with them. This, however, is not our present purpose, and we shall content ourselves by conducting a few inquiries into those portions of the report which more especially apply to medical evidence, as regards the existence or non-existence of insanity. Our space, however, obliges us to defer doing so until the publication of our next.—*Prov. Med. Journ.*

